# Lipofibromatous hamartoma of the median nerve: A case report and review of the literature

**DOI:** 10.4103/0970-0358.53024

**Published:** 2009

**Authors:** V. S. Patil, Sunila Nagle

**Affiliations:** Chief Hand Surgeon-Hand Clinic, India; 1 Prof. Pathology Talegoan Dabhade Med. College, India

**Keywords:** Carpal tunnel syndrome, Lipofibromatous hamartoma, Median nerve

## Abstract

A case of lipofibromatous hamartoma of the median nerve in an adult is described in this article. A 33-year-old male presented with tingling, numbness and swelling in the palm of the left non dominant hand that had been present for a few months. Examination revealed that there was fullness in the volar aspect of the wrist and in the inter-thenar area. Another mass was present at the base of the index finger, which appeared to be involving subcutaneous tissues. The clinical diagnosis was carpal tunnel syndrome due to a space occupying tumor mass in the carpal tunnel. On exploration of the carpal tunnel, a large median nerve was seen 4 cm proximal to the wrist crease line and extending distally until it divided into its branches. Another mass was present at the base of the index finger, which was adherent to the skin. The radial digital nerve of the index finger was normal in size. For the enlarged nerve, an epineurotomy was performed and a biopsy was taken. Another biopsy was taken from the distal mass. His postoperative period was uneventful. In July 2004, at the end of 5 years, the patient had no symptoms and the size of the tumor had not increased.

## INTRODUCTION

Lipofibromatous hamartoma is a rare neoplasm that most commonly involves the median nerve. A review of the literature showed that 88 cases have been reported out of which 33 had macrodactly. Involvement of the ulnar radial and plantar nerves has also been described.[1–4] The tumor is usually present at birth and may be associated with digital enlargement.[5] Treatment of this neoplasm has been controversial. Some authors recommend excision of the involved nerve, some prefer microsurgical intraneural dissection of neoplastic elements, and others advise no treatment or minimal treatment.[1] We present one such case of a patient who was treated with carpal tunnel release, epineurotomy and biopsy and was on follow-up for 5 years.

## CASE REPORT

A 33-year-old right-handed male was seen for evaluation of swelling in front of the left wrist and palm and intermittent tingling in the thumb, index finger, and middle finger that was noticed by the patient 6 months prior to seeking advice. Though the swelling had been present for a few years, the onset of tingling drew his attention.

**Figure 1:**
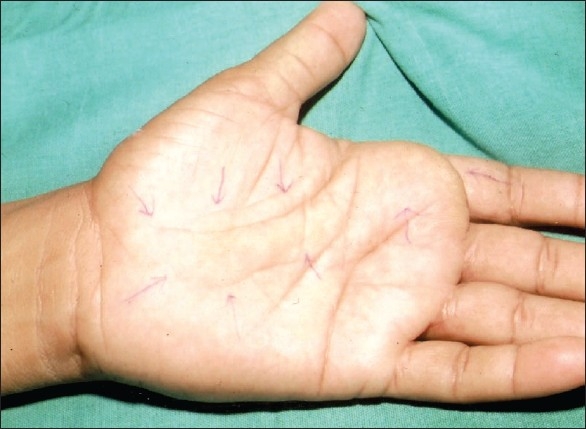
Preoperative photograph showing fullness in the centre of the proximal palm and another mass at the base of the index finger of the left hand

Upon examination, there was fullness over the wrist and in the interthenar area of the palm [Figure 1]. Another swelling was at the base of the index finger. It was soft, non pulsatile, non compressible, and tender to touch. Tinel's sign was positive over the wrist swelling. There was no thenar wasting or sensory deficit. A radiological examination was normal. An ultrasound examination revealed a soft tissue oblong lump extending from the wrist to the palm and another separate mass at the base of the index finger. Nerve conduction studies were not performed.

**Figure 2:**
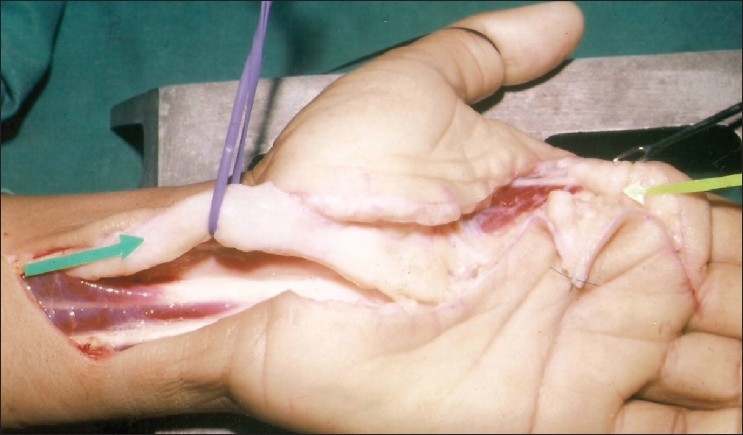
Intraoperative photograph showing a large median nerve in the distal forearm and carpal tunnel, normal sized radial digital nerve of the index finger, and a fibrofatty mass at the base of the index finger

**Figure 3:**
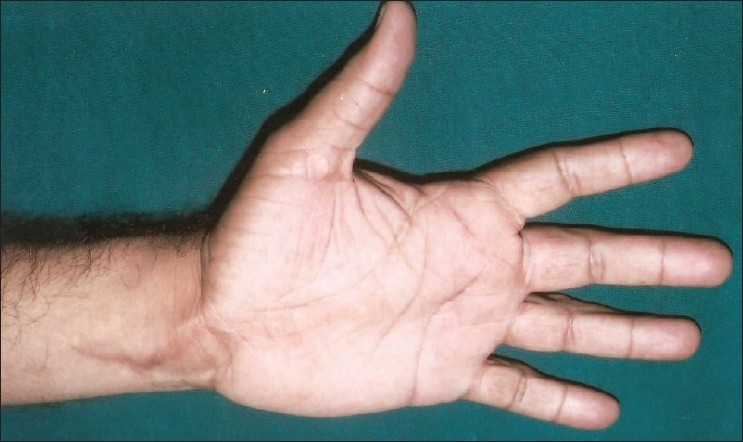
After 5 years, no increase in the size of the tumor

Surgical exploration was performed in February 1999 under brachial block anesthesia with an upper arm tourniquet. The median nerve was found to be grossly enlarged from 4 cm proximal to the wrist crease line to the level of its division into distal branches in mid palm. Common digital nerves were not involved in the thickening. The radial-side digital nerve of the index finger was found to be merging with another fibrofatty mass at the base of the digit [Figure 2]. Carpal tunnel release and epineurotomy of the enlarged median nerve were Performed. A biopsy was obtained from the enlarged median nerve and the fibrofatty mass at the base of the index finger. The latter was only partially debulked for fear of damaging the digital nerve. We did not use intraoperative nerve stimulation. The wound was closed after placement of a small suction drain. The drain was removed on Day 3 and the sutures were removed on Day 14. The patient was kept on a regular follow-up schedule. Even after 5 years, the patient revealed no tingling in the thumb, index finger, and middle finger. He was able to carry out all his routine work. Upon examination, Tinel's sign was negative. Sensations were normal in the index finger and the swelling remained unchanged in size [Figure 3].

### Histopathology

The epineurotomy specimen with the biopsied strip of tissue showed minimal hypertrophy of nerve fibers. It was closely associated with a noncapsulated fibrolipomatous hamartoma, composed of a haphazard mixture of mature fibrous tissue, adipose tissue, a few blood vessels, and occasional small mature nerve twigs. The debulked mass from the base of the index finger also revealed identical features as seen in the enlarged median nerve proving both lesions to be lipofibromatous hamartoma.[1] This mass was infiltrating the subcutaneous tissue resected along with the lesion. There was no neural hypertrophy or true macrodactly.

## DISCUSSION

Swelling involving the median nerve in the region of the wrist is uncommon.[6] True lipomas of the median nerve are rare.[7] They are soft encapsulated tumors that are easily shelled out from the surrounding tissue and contain no neural elements. Hamartomas may be associated with macrodactly[8] and probably present proximal extension of the same regional growth disturbance (macrodystophia lipomatosa). Nerve abnormalities with true macrodactly have been reported so commonly that it led Kelikian to conclude that the most common variety of macrodactly should be called nerve territoryoriented macrodactly (NTOM).[8] The histological nature of the tumor appears to be the same whether or not macrodactly is present.[1]

A developmental or congenital origin appears likely and the tumor may also be classified as a hamartoma since the fibrous fatty and neural elements in the tumor mass are haphazardly distributed but essentially mature. These are localized within the nerve sheath and do not involve surrounding tissues.[9] Although known by various pseudonyms such as fibro-fatty proliferation, intraneural lipoma, unusual tumor of the median nerve, and lipomatous hamartoma, the term lipofibroma best describes the nature of this tumor.[6] The term lipofibromatous hamartoma was introduced by Johnson and Bonfiglio[10] in which there is a localized interstitial fatty infiltration of a nerve with extension into its branches. The subcutaneous tissues are characteristically not involved in this process and there is no cutaneous or skeletal overgrowth. In some cases, a high-degree of clinical correlation between macrodystrophia lipomatosa and lipofibromatous hamartoma may exist.[11,12] Our case probably fits into this category because there is no continuity between two lesions and the lesion at the base of the index finger is a suppressed form of macrodactyly where there is involvement of subcutaneous tissues without overgrowth of bones and overlying skin.

Although the exact cause is unknown, a congenital or developmental origin is likely since the majority of these tumors present and manifest themselves in the young.[9] It has been suggested[13] that pressure from an abnormally developing flexor retinaculum causes their development, although there is little evidence for this and lipofibromas have been described in nerves that are normal within the carpal tunnel.[9] The tumor may occur in the median nerve in the distal forearm, the carpal tunnel, the palm of the hand, or the digits. Patel, *et al.* are of the opinion that the tumor starts proximally in the forearm or distally in the palm.[9] It is when the tumor advances into the carpal tunnel that symptoms of compression neuropathy appear, if at all. They have reviewed the literature and added two cases of their own where the tumor was present since childhood favoring the theory of the congenital nature of these hamartomas.

A diagnosis is usually made from the patient's history and clinical examination. A computed tomography (CT) scan or magnetic resonance imaging (MRI) may be of some help in the diagnosis.

The surgical management of lipofibromatous hamartoma is controversial.[14] Recommendations for treatment of this lesion include decompression of carpal tunnel,[12,15,16] decompression and debulking of fibro-fatty sheath,[17] microsurgical dissection of neural elements,[11] and the excision of the involved nerve segment with or without grafting.[5,18,19]

In our case, decompression was done by dividing the flexor retinaculum and deep fascia in the lower fourth of the forearm, an epineurotomy and biopsy were performed in the carpal tunnel area, and partial debulking of the mass was done at the base of the index finger for biopsy. In between these lesions, a radial-side digital nerve appeared unaffected by the lesion thus ruling out the possibility of the extension of the lesion by continuity from distal to proximal or vice versa.[8,9] At the end of 5 years, he was free from his neurological symptoms and the size of the tumor had not increased.

This procedure of releasing the transverse carpal ligament with or without an epineurotomy has been the most frequently reported operative procedure for this soft tissue tumor.[9,12,16,20] The bulk of the tumor may be reduced using microsurgical techniques[5,11,21] and if small, can be removed without damage to the nerve. If the tumor is big, debulking is time-consuming and certainly compromises the vascularity of the nerve and provokes an intense healing response that may further jeopardize function.[20] In another case report,[14] interfascicular dissection of the tumor resulted in a permanent loss in sensibility and motor function. Lance *et al*. have proposed electromyographic studies to exclude the existence of Martin-Gruber anastomosis before a decision regarding an operation is made.[14] The importance of this variation is also evident in Case 2 of Patel, *et al.* wherein an excision of the entire enlarged nerve in the case of mesenchymoma in a six-year-old male patient had minimal sensory loss after 16 years.[9] In another case of a nine year-old female patient, after a total excision of the mass, there was no serious sensory or motor loss.[19] The result was attributed to the presence of Martin-Gruber anastomosis.

Thus, the radical resection of the involved portion of the nerve would seem to be unnecessary except in children up to the age of two because of the remarkable propensity towards re-education of sensibility and in patients with a definite diagnosis of malignancy or a rapidly growing tumor preferably after EMG studies.[5,18,22,23] If immediate nerve grafting could be performed, a complete resection of a benign bulky tumor like angiolipofibroma or lipofibroma is worth considering.[21]

Our case highlights the widely accepted modality of surgical treatment, namely the release of carpal tunnel and partial debulking of the separate mass at the base of the index finger, which has given symptomatic relief, and there is no increase in the size of the tumor after 5 years. Through this case report, we question the expostulation of Patel *et al*. as pertains extension of the lesion proximally or distally by continuity. It is most likely a clinical coexistence of lipofibromatous hamartoma and macrodystrophia lipomatosa[11,12] with no continuity.
